# Annual Meeting of Governors

**Published:** 1920-06-26

**Authors:** 


### ST. THOMAS'S HOSPITAL.

### Annual Meeting of Governors.

The Annual Meeting of the Governors of St. Thomas's Hospital was held on Wednesday, June 23. The Treasurer, the Hon. Sir Arthur Stanley, C.B., presided. The accounts for the past year were put before the Governors. These showed that the hospital is in a serious financial position. The sum of £105,739 was owing to the bankers at the beginning of the year. This position is largely due to the heavy increase in expenditure, and is due to the accumulation of deficits during the latter years of the war. The Governors have every reason to be proud of the work undertaken by the hospital and its staff, for a total of 11,376 military patients were treated in the hospital during the war. Of these, 1,489 were officers and 9,887 were N.C.O.s and men. As an instance of the heavy increase in cost, the Treasurer pointed out that the cost per patient per day was 7s. in 1914. It rose to 7s. 9¾d. in 1918. In 1919, however, the cost was abnormally high—13s. 6½d. per patient per day. This was owing to the fact that up to March 1919 St. Thomas's was a military hospital, but demobilisation of the hospital had begun and many of the wards were not kept full in patients. The cost of running was, however, practically the same as when full, except for food, because the staff had to be maintained even though patients were being demobilised. As soon as the military patients were gone, extra cost became involved of fitting the hospital for civilian purposes. This accounted for the very high cost in 1919. It must, however, be recognised that there is a very serious permanent rise in the cost of maintaining the hospital, owing to the high price of provisions, increased cost of all materials, the great increases in the cost of drugs, dressings, and the abnormal increase in wages which are now being paid.

### Future Prospects.

Sympathy was expressed with the extraordinary good work carried out by the nurses, and the Treasurer stated that, with the present increase in the rates paid, salaries of nurses will amount to over £10,000 per year, as against £6,750 for salaries in 1914. Another heavy item in the expense of a modern hospital is the development of the social work under the charge of the lady almoner. Having had fifteen years' experience of this work, St. Thomas's Hospital is enthusiastic in its favour, and feels that the almoner's work is necessary to make skilled treatment effective. Whilst the expenditure must remain very high, there is no definite prospect of an increase in income. About half the amount required is received from the endowments of the hospital, and a strong appeal is therefore made for subscriptions and donations, and especially for legacies. It is interesting that in the last five years the average receipt from legacies has been £3,660. In the previous five years the average receipts from legacies was £19,272 per annum, while in the five years previous to that the receipts from legacies averaged £22,870. St. Thomas's Hospital is one of the oldest hospitals in the kingdom, and proud of its great traditions; but if it is to carry out its work effectively it must have the support of the public, and for this a most earnest appeal is now made.

